# Ultrasound-Induced Structural Modification of Cellulose in Poplar Wood: Effects on Crystallinity and Enzymatic Hydrolysis Efficiency

**DOI:** 10.3390/ma18225156

**Published:** 2025-11-13

**Authors:** Monika Marchwicka, Eva Výbohová, Andrzej Radomski

**Affiliations:** 1 Department of Wood Science and Wood Protection, Institute of Wood Sciences and Furniture, Warsaw University of Life Sciences in Warsaw, Nowoursynowska 166 Street, 02-787 Warsaw, Poland; andrzej_radomski@sggw.edu.pl; 2 Department of Chemistry and Chemical Technologies, Faculty of Wood Sciences and Technology, Technical University in Zvolen, T. G. Masaryka 24 Street, 960-01 Zvolen, Slovakia; vybohova@tuzvo.sk

**Keywords:** cellulose, crystallinity, ultrasonic treatment, enzymatic hydrolysis, poplar wood, biomass materials, bioethanol

## Abstract

**Highlights:**

**What are the main findings?**

- Ultrasound did not increase glucose yield after enzymatic hydrolysis of raw wood.
- Short-term ultrasound may enhance early hydrolysis of cellulose.
- Prolonged ultrasonication reduced enzyme activity and final glucose yield.
- Cellulose crystallinity was not significantly altered by ultrasound.

**What are the implications of the main findings?**

- Ultrasound alone is ineffective for saccharifying untreated woody biomass.
- Enzyme deactivation limits the benefits of long ultrasonication.
- Process optimisation is key for ultrasound integration in biorefineries.

**Abstract:**

The crystalline structure of cellulose plays a crucial role in its reactivity, which is particularly important in biomass processing involving enzymatic hydrolysis. This study investigates the effect of low-frequency ultrasound (40 kHz) on the efficiency of enzymatic hydrolysis of cellulose in poplar wood and its structure, with a focus on its crystallinity and susceptibility to enzymatic hydrolysis. Two experimental pathways were explored: ultrasound-assisted hydrolysis of raw wood and isolated cellulose. Structural modifications were assessed using Fourier-transform infrared spectroscopy to determine the Lateral Order Index (LOI) and Total Crystallinity Index (TCI), providing insight into the reorganisation of cellulose microstructure. To evaluate hydrolysis efficiency, glucose yields were quantified by high-performance liquid chromatography. The application of ultrasound to raw wood resulted in minimal improvement in sugar release, whereas pretreatment of extracted cellulose led to a modest acceleration of enzymatic hydrolysis. The results show that ultrasound-assisted hydrolysis of raw wood did not significantly increase glucose yield, reaching only 9.9 ± 0.3% and 10.1 ± 0.8% for two poplar hybrids. Only slight increases in TCI and no significant changes in LOI were observed after 4 h of ultrasonic exposure. It indicates limited disruption of the crystalline structure under the tested conditions.

## 1. Introduction

Turning plant material and waste into fuel is a challenge. As the world seeks sustainable alternatives to fossil fuels, bioethanol stands out as a promising solution. Bioethanol derived from renewable plant materials can lower dependence on fossil fuels and reduce greenhouse gas emissions [1]. Enzymatic hydrolysis is the process of breaking down cellulose, the main component of plant biomass, into simple sugars using cellulase enzymes. Enzymatic hydrolysis is considered more eco-friendly than other methods, such as acid hydrolysis. Some of its benefits include lower energy requirements, reduced equipment corrosion, high specificity with fewer by-products, reduced chemical usage, and the fact that enzymes are biodegradable [2]. With the proper selection of conditions and the use of adequate material pretreatment, an economically efficient process can be achieved [3]. However, despite its advantages, hydrolysing cellulose, especially from woody materials, is not easy. Due to the difficult accessibility of cellulose in lignocellulosic biomass, the bioethanol production process involves several steps. The first step is to harvest raw material with a high cellulose content. The raw material is then subjected to pretreatment to increase the overall process efficiency [2,4]. Enzyme access to polysaccharides is enhanced by increasing porosity, altering the chemical composition, or modifying the properties of the individual components. Enzymatic hydrolysis of the pretreated material is then carried out. The consequence is the production of simple sugars, which are then fermented into ethanol. In the final step, the alcohol is distilled and dehydrated to produce a liquid transport fuel [3,5,6].

The enzymatic hydrolysis of cellulose present in the wood is a process influenced by many factors. They can be divided into those related to the mechanism of enzyme action and those related to the raw material. This classification is one of many proposed, and it can sometimes be difficult to classify a factor into only one group. Due to the variability of cellulolytic enzymes and the heterogeneous structure and composition of wood, it is challenging to understand the interaction between the enzyme and wood fully [7]. Additionally, this relationship evolves as the hydrolysis process progresses. Among the most crucial wood properties affecting this process are wood porosity, lignin and hemicellulose content, cellulose crystallinity degree, and presence of inhibitors and acetyl groups [8,9,10]. Cellulose crystallinity refers to the ordered arrangement of cellulose molecules within the polymer. Lower crystallinity enhances the accessibility of cellulose to chemical and enzymatic reactions, which is critical for processes such as enzymatic hydrolysis [11]. Wood is a raw material rich in sugars, but exploiting this potential requires more time and energy than pure cellulose. Due to the low efficiency of enzymatic hydrolysis of cellulose in wood, which is hindered by numerous obstacles, various methods to enhance this efficiency and accelerate the enzymatic hydrolysis of cellulose in wood are being investigated [12,13,14,15,16,17,18,19]. Wood continues to be investigated as a bioethanol feedstock due to its widespread availability, high carbohydrate content, and non-edible nature. It does not have to compete with other feedstocks used for bioethanol production, as it is important to have diverse feedstock sources to ensure flexibility, resilience, and long-term sustainable biofuel production [20]. Improving processing efficiency for wood can reduce enzyme requirements and production costs, supporting the economic viability of bioethanol. Continued research on wood feedstocks is therefore essential for advancing sustainable lignocellulosic biofuel production [21,22,23].

Pretreatment of lignocellulosic biomass using ultrasound to increase the efficiency of enzymatic hydrolysis is a new developing technology. Its use in combination with other pretreatment methods [21] or during enzymatic hydrolysis is also investigated [24,25]. During ultrasonic treatment, the phenomenon of cavitation occurs. Ultrasonic waves propagating through the liquid produce areas of low or high pressure. If the pressure drops below the vapour pressure, water evaporates, and cavities (bubbles) form. The water vapour bubbles can rise to the surface or, if the pressure exceeds the water vapour pressure, they implode (collapse) [26]. Cavitation involves the generation, growth, and implosion of cavities, resulting in a shock wave, pressures reaching up to 1000 atm, and local temperatures that can rise to around 4700 °C. Free radicals are produced during bubble implosion. Cavitation occurs when the cavitation threshold is exceeded, i.e., a specific value of ultrasound intensity that depends on the type of liquid, the frequency of the wave, and the presence of impurities in the liquid [27]. The effect of ultrasound on woody biomass causes an increase in porosity, and what is interesting from the viewpoint of enzymatic hydrolysis is the increase in the share of macro and mesopores that are of sufficient size to be accessible to enzymes (macropores and larger mesopores) [28]. The use of ultrasound during enzymatic hydrolysis also results in the transfer of enzyme molecules from the liquid phase to the biomass (solid phase) and the hydrolysis reaction products (sugars) to the liquid phase [24]. Water, together with increasing temperature and ultrasound (the cavitation), can cause swelling in the cellulose, thus decreasing its crystallinity. On the other hand, the amorphous, less resistant areas of the cellulose fibres are more easily degraded, thereby increasing the content of the crystalline regions in the entire material. Both described phenomena are desirable in the enzymatic hydrolysis of cellulose, due to easier access of enzymes to the cellulose. The use of ultrasound in the pretreatment of lignocellulosic materials increases the amount of sugars obtained from enzymatic hydrolysis and thus increases the efficiency of the bioethanol production process [29,30]. According to Wang et al., ultrasonic treatment breaks the α-O-4 and β-O-4 ether bonds in lignin. Consequently, the biomass is degraded due to the splitting of the lignin and polysaccharide components of the lignocellulosic biomass [31]. It has been reported that the ultrasonic pretreatment (20 kHz, sonic power 0.16 kW/L, 1 h) of rice straw caused the lignin to split and the surface of the cellulose to become visible. Rice straw was also treated with an ultrasound at 20 kHz and sonic power of 0.16 kW/L during the enzymatic hydrolysis process (50 °C, pH of 4.8). When compared to untreated rice straw, the glucose concentration in treated rice straw increased significantly by 57.65% [32]. It has also been proven that applying a 50 kHz ultrasonic at 50 °C significantly increases enzyme efficiency during the enzymatic hydrolysis of corn stover and sugar cane bagasse [33]. Revin et al. investigated that crushed *Pinus sylvestris* wood presonicated for 20 min at a power of 10 W/kg after enzymatic hydrolysis results in a glucose yield that is up to 60% of the theoretical maximum [34]. All these investigations applied low-frequency ultrasound. It has been proven that during the application of low-frequency ultrasound, most of the energy is consumed by the cavitation process. As explained above, cavitation is the desired phenomenon during both pretreatment and enzymatic hydrolysis of lignocellulosic biomass. Enzymatic hydrolysis plays an important role in converting cellulose into valuable products, particularly biofuels. Each type of lignocellulosic biomass differs in its exact chemical composition, including the content of lignin and polysaccharides, as well as the structure of lignin. Therefore, methods used on different types of biomass, although promising for one type, may not always be effective on another. It is worth examining how methods used to increase the glucose yield of lignocellulosic materials, such as rice straw, corn stover, and sugar cane bagasse, will affect the efficiency of enzymatic hydrolysis of the cellulose contained in poplar wood.

Given the obstacles that are met during the enzymatic hydrolysis of cellulose in wood and the resulting low glucose yield, this study investigated the effect of ultrasound on glucose yield after enzymatic hydrolysis of cellulose contained in wood. Ultrasound was applied during the enzymatic hydrolysis process with no pretreatment of the wood. In this study, low-frequency ultrasound was used. Literature data support this decision, which states that during the application of low-frequency ultrasound, most of the energy is consumed by the cavitation process, and it is cavitation that is the desired phenomenon during enzymatic hydrolysis [29,33,35]. Low-frequency ultrasound covers a range of up to about 100 kHz. The ultrasonic cleaner used in this study produced waves at 40 kHz. The effect of ultrasound at this frequency on enzyme activity and its impact on glucose yield after enzymatic hydrolysis of cellulose extracted from wood was also investigated to gain a better understanding of the overall process. It was also investigated how ultrasound at a given frequency affects the Total Crystallinity Index (TCI) and Lateral Order Index (LOI) of cellulose obtained from wood. Although ultrasound-assisted enzymatic hydrolysis has shown promise for agricultural residues, its effectiveness on woody biomass, such as poplar wood, is less understood. This study provides important insights by showing that low-frequency ultrasound has a limited impact on cellulose crystallinity and glucose yield in raw wood and extracted cellulose. These results highlight the boundaries of ultrasound as a single pretreatment method. Knowing these limitations can help with future process optimisation and also avoid inefficient applications. It is important for more sustainable bioethanol production strategies.

## 2. Materials and Methods

### 2.1. Materials

Wood from the trunk of *Populus deltoides* × *maximowiczii*, a 5-year-old tree, and *P. trichocarpa*, a 3-year-old tree, was used in the study. The native (non-GMO) wood was obtained from the experimental field WOLICA, Department of Plant Genetics, Breeding and Biotechnology, Warsaw University of Life Sciences (52°08′59.1″ N 21°03′51.6″ E). The obtained material was seasoned for six months in the laboratory. Then, it was debarked and milled using a Retsch SM100 laboratory mill (Retsch GmbH, Haan, Germany). A fraction of sawdust (<0.43 mm) was used for the investigation. 5-year-old poplar wood *P. deltoides* × *maximowiczii* was composed of (1.6 ± 0.1)% extractives soluble in chloroform–ethanol (93:7_*w*/*w*_), (82.3 ± 0.4)% holocellulose, (52.0 ± 0.3)% cellulose, and (22.1 ± 0.3)% lignin. 3-year-old poplar wood *P. trichocarpa* was composed of (1.5 ± 0.1)% extractives soluble in chloroform–ethanol (93:7_*w*/*w*_), (51.8 ± 0.3)% cellulose, (85.9 ± 0.2)% holocellulose, and (20.3 ± 0.3)% lignin.

Cellulose obtained from 3-year-old poplar wood *P. trichocarpa* by the Kürschner-Hoffer method (3 heating cycles in a mixture of nitric acid and ethanol were applied) was also used in the study [36].

The following reagents were used in this study: ethyl alcohol (96%, denatured with diethyl ether and tert-butyl methyl ether, Linegal Chemicals (Warsaw, Poland), technical grade), sodium azide (CHEMPUR (Karlsruhe, Germany), p.a. grade), sodium citrate (CHEMPUR, p.a. grade), glucose (Sigma-Aldrich (St. Louis, MO, USA), p.a. grade), nitric acid (65%, CHEMPUR, pure grade), citric acid (CHEMPUR, p.a. grade), and sodium hydroxide (CHEMPUR, pure grade).

### 2.2. Ultrasound-Assisted Enzymatic Hydrolysis of Cellulose in Wood

For enzymatic hydrolysis, it was necessary to determine both the cellulose content and the moisture content of the material. A mass of material corresponding to 100 mg of the dry weight of cellulose was placed in a 10 cm^3^ glass test tube. Then, 5 cm^3^ of citrate buffer pH 4.8, 0.1 cm^3^ of 0.1 M sodium azide solution, 100 mg of 25% Cellic^®^ CTec2 enzyme blend solution (Novozymes, Davis, CA, USA), and distilled water were added to the tube to achieve the desired volume. The tubes were placed in an ultrasonic cleaner at 50 °C for 72 h. The investigations were conducted using a POLSONIC Ltd. (Warsaw, Poland) ultrasonic cleaner, model Sonic-3, with a frequency of 40 kHz and an ultrasonic power of 2 × 160 W. The water from the ultrasonic cleaner was recirculated through a cooler to maintain a constant water temperature in the bath. The temperature of the bath water was monitored during the process. After 72 h of enzymatic hydrolysis, samples were collected and stored frozen at −24 °C until HPLC analysis.

### 2.3. Ultrasound-Assisted Enzymatic Hydrolysis of Cellulose Isolated from Wood

Cellulose isolated by the Kürschner-Hoffer method from the 3-year-old poplar wood *P. trichocarpa* was used for this study. Enzymatic hydrolysis was prepared in 10 cm^3^ tubes as described above and then placed in the low-frequency ultrasonic cleaner (40 kHz). Three series of samples were prepared. The first was ultrasonicated for 1 h and then transferred to a water bath at 50 °C. The second was subjected to ultrasonication for 4 h and then also transferred to a water bath. The third series was a reference series that was not ultrasonicated but placed directly in a 50 °C water bath. Samples were taken from tubes after 1, 4, and 24 h. For comparison purposes, enzymatic hydrolysis without ultrasound was carried out in a water bath at 50 °C. Samples for chromatographic analysis were taken after 1, 4, and 24 h. Additionally, the experiment was repeated with different sonication times (0, 0.5, 1, and 4 h), and samples for chromatographic analysis were collected at 1, 4, 24, and 48 h.

### 2.4. Ultrasound Treatment of Cellulose Isolated from Wood

Cellulose obtained from 3-year-old poplar tree (*P. trichocarpa*) wood was subjected to ultrasonication at a frequency of 40 kHz and a temperature of 50 °C for 24 h. Reference samples for testing the influence of the temperature were performed without the US, applying only a temperature of 50 °C. The samples for each experiment were collected after 0, 0.5, 1, 2, 4, 8, and 24 h.

### 2.5. Fourier-Transform Infrared Spectroscopy (FTIR)

In this analysis, Kürschner–Hoffer’s cellulose samples were examined using attenuated total reflectance–Fourier-transform infrared spectroscopy (ATR-FTIR, Agilent Technologies, Santa Clara, CA, USA). The measurements were conducted using a Nicolet iS10 FTIR spectrometer (Thermo Fisher Scientific, Waltham, MA, USA) equipped with a Smart iTR ATR sampling accessory featuring a diamond crystal from Thermo Fisher Scientific in Madison, WI, USA. The instrument settings were as follows: a resolution of 4 cm^−1^, 32 scans for each sample, and a wavenumber range from 4000 to 650 cm^−1^. Four analyses were performed on each sample. To analyse the obtained spectra, OMNIC 8.0 software from Thermo Fisher Scientific was used. The Lateral Order Index (LOI) was determined by calculating the ratio between the band at 1429 cm^−1^ (C–H wagging) and the band at 898 cm^−1^ (asymmetric out-of-phase ring stretch in the C1–O–C4 glycosidic linkage) [37]. The Total Crystalline Index (TCI) was calculated by taking the ratio between the bands at 1368 cm^−1^ (C–H bending) and 2900 cm^−1^ (C–H stretching) [38]. Peak heights were measured for calculating the TCI and LOI. The ATR correction and baseline correction were performed on the obtained spectra.

### 2.6. Enzyme Activity

Cellic^®^ CTec2 enzyme blend solution (25%) was subjected to ultrasonic treatment for 4 h. Then, the activity of the enzyme blend was determined. The activity of the enzyme blend without pretreatment was defined as a reference sample. To exclude the influence of the temperature used during the process, the activity of enzymes heated for 4 h in a water bath at 50 °C was also examined. The NREL procedure expressing enzyme activity in FPU was used to determine activity [39].

### 2.7. HPLC Analysis

The glucose content in the hydrolysate was determined using a Shimadzu liquid chromatograph (Shimadzu, Kyoto, Japan) with a refractometric detector. A dedicated Rezex™ RHM-Monosaccharide H+ column from Phenomenex (Torrance, CA, USA) was used for the analysis. Analytical conditions: eluent—redistilled water, flow rate 0.6 cm^3^/min, oven temperature 80 °C. Chromatograms were analysed using LC Solution v.1.21 SP1 software.

Based on the analysis of a series of glucose standard solutions of known concentration, a calibration correlation was prepared to quantify the glucose obtained in the hydrolysates. Calibration correlation:

$$C=(3.654\pm 0.013)\times 10^{-7}A$$

where

C—glucose concentration/(mg/cm^3^);
A—peak area.

The mass of glucose in the blank sample (initial) was also included.

### 2.8. Calculation of Glucose Yield After Enzymatic Hydrolysis

For the material after each treatment method, the cellulose content was determined using the Kürschner–Hoffer method. Based on this content, the mass of cellulose in the sample was determined. The theoretical mass of glucose (TMG) was calculated by multiplying the mass of cellulose in the sample by the resulting factor of 1.11, which corresponds to the ratio of the mass of glucose (C_6_H_12_O_6_—180.16 u) to the mass of the glucopyranose unit (C_6_H_10_O_5_—162.14 u). The percentage of glucose yield after enzymatic hydrolysis was calculated as a ratio of glucose mass (obtained in the hydrolysate) to the TMG of the tested samples.

## 3. Results and Discussion

### 3.1. Ultrasound-Assisted Enzymatic Hydrolysis of Wood

Initially, ultrasound-assisted enzymatic hydrolysis of wood was performed. Considering the complex structure of wood and the difficult accessibility of cellulose within it, it was decided to use ultrasound throughout the entire duration of the enzymatic hydrolysis, which was conducted for 72 h. The 72 h conditions for ultrasound-assisted enzymatic hydrolysis were selected based on preliminary experiments and previous studies, which demonstrated that this duration allows sufficient time to achieve stable hydrolysis efficiency for wood biomass.

The obtained results of glucose yield after enzymatic hydrolysis of two poplar wood species—3-year-old *P. trichocarpa* and 5-year-old *P. deltoides* × *maximowiczii*—using ultrasound are summarised in Table 1. Reference samples of enzymatic hydrolysis without ultrasound were also carried out for the used materials. Due to the rigid structure of wood and the limited access to cellulose, largely shielded by lignin and hemicelluloses, ultrasound was hypothesised to enhance enzyme penetration and activity. However, as shown in Table 1, glucose yields for both *P. trichocarpa* (3-year-old) and *P. deltoides* × *maximowiczii* (5-year-old) were not improved by ultrasound treatment compared to the control. Despite the anticipated benefits of cavitation and acoustic streaming on mass transfer, the data reveal that ultrasound, under these conditions, does not overcome the physical barriers posed by the native wood matrix. Based on the results, no increase in glucose yield was obtained after enzymatic hydrolysis of wood using ultrasound compared to the reference sample. This observation is evident for both of the poplar wood species used. Glucose yield after 72 h of enzymatic hydrolysis of 5-year-old *P. deltoides* × *maximowiczii* poplar wood was slightly lower with ultrasound, and this difference was statistically significant. This effect may result from partial inactivation of the enzymes caused by ultrasonication. In the absence of dissolved gas, ultrasound can induce cavitation, producing shock waves that disrupt macromolecules, including enzymes. Such enzyme inactivation likely contributes to the reduced glucose yield observed after prolonged ultrasonic exposure.

Several studies have investigated the potential of ultrasound to enhance the enzymatic hydrolysis of biomass. However, the outcomes vary significantly depending on the substrate type, pretreatment method, and ultrasound parameters. Ultrasound has been shown to enhance enzymatic hydrolysis, particularly in pretreated or agricultural biomass. Studies have reported significant improvements in glucose yield for rice straw, corn stover, and sugarcane bagasse [21,24]. More efficient was applying ultrasound-assisted hydrolysis with simultaneous other pretreatments of corn stover, rice straw, and sugarcane bagasse, typically after chemical or mechanical pretreatment, which has shown significant improvements in glucose yield due to better enzyme accessibility and enhanced mass transfer [40,41,42,43]. Another example is a wood-chip residue (sawdust) containing equal proportions of hardwood (beech) and softwood (fir), subjected to simultaneous ultrasound-assisted alkaline pretreatment due to low efficiency of glucose yield [44]. These findings align closely with our results, suggesting that in biomass, also in raw woody biomass, the protective lignin–hemicellulose matrix remains a major limiting factor, one that ultrasound alone, especially under mild, non-destructive conditions, cannot sufficiently overcome. Therefore, while ultrasound has shown promising results in enhancing the enzymatic hydrolysis of certain types of biomass, particularly agricultural waste or pre-treated materials, its solo effectiveness on native wood appears to be limited by its very nature.

### 3.2. Ultrasound-Assisted Enzymatic Hydrolysis of Cellulose Isolated from Wood—Cellulose as the Model Substrate

To better understand the lack of effect of ultrasound on the enzymatic hydrolysis of wood, the enzymatic hydrolysis was repeated using cellulose isolated from wood by the Kürschner-Hoffer method as the model substrate. Cellulose, as a material for enzymatic hydrolysis, was used to eliminate the influence of other factors on the process, such as the presence of lignin and hemicelluloses that block access to cellulose.

**Figure 1:**
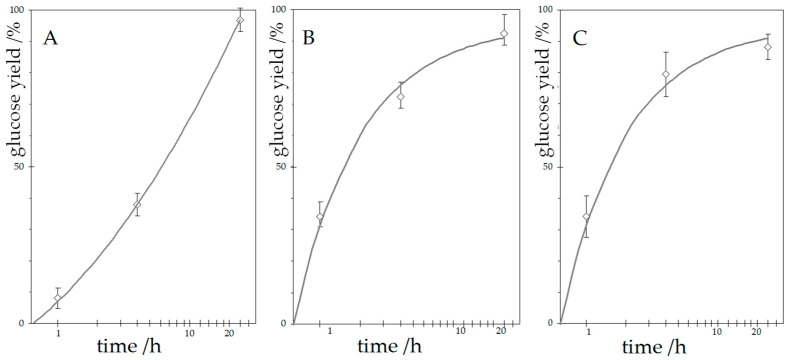
Glucose yield after enzymatic hydrolysis with and without ultrasonic irradiation of cellulose (sonication time: left (**A**)—0 h, middle (**B**)—1 h, right (**C**)—4 h).

Figure 1 highlights a significantly different kinetic profile for glucose yield after enzymatic hydrolysis with and without ultrasonication. For samples not treated with ultrasound, hydrolysis followed an atypical trajectory for chemical reaction. Unusually for chemical reactions, the rate of the process increases over time, suggesting that the rate-limiting factor for the entire process is not the chemical reaction rate, which, apart from autocatalytic processes, typically decreases over time or remains constant. Such a trend contradicts the typical behaviour of chemical reactions, where rates decline as reactants are consumed. This observation suggests that diffusion limitations, rather than the enzyme’s internal catalytic activity, dictate the process rate. It can be concluded that the rate-limiting factor of the entire process is the diffusion of enzyme molecules within the fibres and the availability of cellulose to their action. In this case, at the initial stage, the enzyme acts on a relatively small area, and the rate of the process is slow. During the process, as a result of swelling and degradation of the cellulose chains, the available surface area increases, which increases the total rate of the process.

In contrast, samples exposed to ultrasound showed enhanced glucose yields in early stages. Just one hour of ultrasound-assisted enzymatic hydrolysis increased glucose yield. The use of ultrasound in the initial stage of the process had a beneficial effect on the glucose yield after enzymatic hydrolysis. This confirms that ultrasound enhances initial enzyme access to the substrate, likely by disrupting the fibre surface or improving mass transport. According to the data shown in Figure 1, the glucose yield after 1 h of enzymatic hydrolysis of cellulose with ultrasound (Figure 1B,C) was very similar (only slightly lower) than the yield obtained after 4 h of enzymatic hydrolysis without ultrasound (Figure 1A). The glucose yields after 4 h of enzymatic hydrolysis with ultrasound applied for 1 h and 4 h differed by about 10% in favour of the process with longer ultrasonication. However, after 24 h, the trend reversed: glucose yields were higher in samples that were hydrolysed without ultrasound. This paradox points to a deleterious effect of prolonged ultrasound exposure on enzyme integrity. After 24 h of enzymatic hydrolysis, the glucose yields with ultrasonication applied for 1 h and 4 h were 5 and 9% lower, respectively, compared to the yields obtained for the material hydrolysed without assistance. This could have resulted from the reduced enzyme activity caused by ultrasonication. When exposed to ultrasound, if there is no dissolved gas in the water, a cavitation phenomenon can occur, causing a shock wave that degrades macromolecules, including the enzyme, inactivating it. The reduction in enzyme activity may explain the decrease in glucose yield after prolonged ultrasonication.

**Figure 2:**
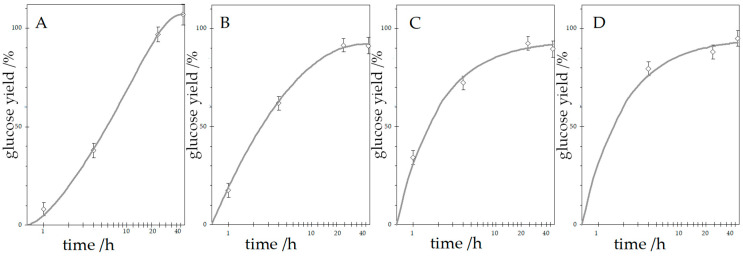
Glucose yield after enzymatic hydrolysis with and without ultrasonic irradiation of cellulose (sonication time: left (**A**)—0 h, middle left (**B**)—0.5 h, middle right (**C**)—1 h, right (**D**)—4 h).

To further evaluate the effect of ultrasonic pretreatment on enzymatic hydrolysis, different sonication times (0, 0.5, 1, and 4 h) were applied during enzymatic hydrolysis. Additionally, the total hydrolysis time was extended up to 48 h to assess how prolonged enzymatic treatment influences glucose yield and whether it can compensate for the effects of sonication on enzyme activity. The results are presented in Figure 2.

The results demonstrate a significant influence of sonication on hydrolysis efficiency. As hydrolysis proceeded, the efficiency increased substantially, with the effect of sonication becoming more pronounced. After 4 h of enzymatic hydrolysis, efficiency ranged from 37.9% (0 h sonication) to 79.5% (4 h sonication), indicating that extended sonication significantly enhances substrate accessibility or removal of inhibitors that arise.

At 24 and 48 h of enzymatic hydrolysis, all samples showed high glucose yield above 88%. The highest value of 106.9% was observed for the cellulose subjected to enzymatic hydrolysis for 48 h without ultrasonication. This can be attributed to complete substrate conversion, resulting in a higher mass of glucose obtained compared to the initial cellulose mass, due to the attachment of a water molecule to the remaining glucopyranose obtained by cleaving the β(1,4)-glycosidic bonds present in cellulose chains. What is interesting is that after 48 h of enzymatic hydrolysis, differences between applied sonication treatment times diminished, suggesting that prolonged hydrolysis time can compensate for the shorter sonication time.

These findings confirm that sonication is beneficial in accelerating enzymatic hydrolysis, particularly in the early phases. Prolonged sonication may negatively affect enzyme activity, which may potentially be through structural changes to the substrate or partial enzyme inactivation. Although extending the enzymatic hydrolysis time to 48 h helped minimise these differences, the efficiency of the sonicated samples remained lower than that of the control (no sonication), confirming that ultrasonication has a lasting inhibitory effect on enzyme performance.

These observations collectively suggest that the timing and duration of ultrasound application are critical. Rather than applying ultrasound continuously, it may be more effective to use it selectively, especially at the beginning of hydrolysis or immediately after pretreatment steps. For example, after steam explosion, alkaline pretreatment, or organosolv processing, ultrasound can help accelerate the removal of soluble inhibitors, such as furfural, HMF, or residual acid by-products, which are known to impair enzymatic performance. This application could enhance both reaction kinetics and final yields by reducing inhibitory feedback and improving enzyme–substrate interaction in a cleaner, more accessible matrix.

### 3.3. Enzyme Stability Under Ultrasound

To probe this further, enzyme activity was measured following different treatments. The activity of the enzyme blend after ultrasonic treatment at 50 °C, which corresponds to enzymatic hydrolysis conditions, was determined. The activity of the enzyme blend without the influence of ultrasound and temperature and the activity after heat treatment in a water bath at 50 °C were also determined. Results are shown in Table 2.

As shown in Table 2, the highest activity was obtained for the non-treated enzyme (129 FPU/cm^3^), and similar activity was observed for the enzyme after heat treatment at 50 °C (127 FPU/cm^3^). When heated to 50 °C for 4 h, the enzyme activity did not change significantly. However, a significant decrease in enzyme activity was seen after ultrasound treatment for 4 h at 50 °C. Prolonged ultrasound exposure at 50 °C significantly reduced enzymatic activity, dropping from 129 to 93 FPU/cm^3^ after 4 h, which is 36 FPU/cm^3^ lower than that for the untreated enzyme. These findings align with prior reports where cellulase activity decreased after ultrasound exposure, especially at high power or prolonged durations. Literature data confirm a decrease in the activity of other enzyme blends containing cellulase under ultrasound treatment, with a simultaneous increase in the efficiency of enzymatic hydrolysis using high ultrasound power. The cellulase solution (from Advanced Biotechnologies, Mumbai, India) was treated for 30 min at various temperatures (20, 30, 40, and 50 °C) using an ultrasonic probe (20 kHz) at 17.33 W/cm^2^, which causes an improvement in enzyme activity at the beginning and then after 60 min of treatment, a decrease in cellulase activity [25]. Other researchers treated cellulase (from Novozymes (China) Biotechnology Co., Tianjin, China) with ultrasound at 15 W and 24 kHz, and observed the lower activity of used enzymes just after 25 min of treatment [35]. Additionally, commercially used cellulase enzyme mixtures (Celluclast 1.5 L) were subjected to ultrasound treatment (frequency of 40 kHz and a power of 500 W), resulting in a decrease in their activity [45].

Potential causes of the activity decrease include protein denaturation, hydroxyl radical formation, and mechanical degradation from shockwaves generated during cavitation. Enzymes are inherently sensitive to their microenvironment and, therefore, are particularly vulnerable to these stresses. Shock waves, high rise in local temperature, and formation of hydroxyl radicals may be limiting factors in the combined use of ultrasound and enzymes. These phenomena may cause a decrease in enzyme activity due to protein degradation or denaturation. Enzymes can be denatured even by subtle changes in environmental conditions, including temperature, pressure, pH, or shear stress [46]. Thus, while short-term ultrasound application accelerates hydrolysis, extended exposure compromises enzymatic functionality, negating initial benefits. Ultrasonication has been found to reduce the final glucose yield. Ultrasonic degradation of the enzyme is responsible for this result. This does not preclude the applicability of ultrasonication during enzymatic hydrolysis, as a slight decrease in efficiency is compensated by a significant increase in the process rate. Therefore, the key question was whether the ultrasound influences the cellulose structure, facilitating the access of enzyme macromolecules, or whether it improves mass exchange processes, facilitating the diffusion of enzymes and hydrolysis products inside the cellulose fibres. For this purpose, changes in cellulose properties during the process were investigated.

### 3.4. Effect of Ultrasound on Cellulose Structure

To determine whether ultrasound alters cellulose structure, we examined crystallinity indices: TCI (Total Crystallinity Index) and LOI (Lateral Order Index), based on FTIR spectra.

The TCI values of cellulose obtained from 3-year-old wood of the poplar tree (*P. trichocarpa*) were investigated after applying a temperature of 50 °C, along with the reference sample. Results are shown in Table 3. During ultrasonication, the mixture was heated, and therefore, two factors—ultrasound and temperature—act on the cellulose. The obtained results showed no significant changes in TCI values of cellulose after temperature treatment at 50 °C. Additionally, the TCI values of cellulose treated with ultrasound and native cellulose (the reference) were investigated. The TCI values of the cellulose after ultrasonication showed an increasing trend with the time of ultrasound application (the highest increase after 4 h). This increase, however, was small and only statistically significant according to a *t*-test after the 4 h mark. In addition, this micro-increase is similar to the increase caused by the action of elevated temperature and water, which was found to be statistically insignificant.

The LOI values of cellulose obtained from 3-year-old wood of the poplar tree *P. trichocarpa* were investigated after treating it with ultrasound and separately at 50 °C, and compared to those of native cellulose (reference). The obtained results are presented in Table 4. There was no statistically significant increase in LOI values with the increasing duration of ultrasound and temperature application. This suggests that the ultrasound treatment did not result in a significant change in the crystallinity of the cellulose. Results in Table 3 and Table 4 reveal no statistically significant changes in the LOI, and only a minor increase in the TCI after 4 h of ultrasonication. These small shifts, comparable to those caused by temperature alone, indicate that ultrasound did not substantially alter cellulose crystallinity. Ultrasound did not break down or reorganise the cellulose structure in a meaningful way. Therefore, the improved hydrolysis rate cannot be attributed to structural modification. Instead, the evidence suggests that enhanced mass transfer is the primary mechanism. Ultrasound likely facilitates increased enzyme diffusion into cellulose micro- and nano-pores, as well as more efficient removal of hydrolysis products (e.g., glucose, cellobiose), which can otherwise inhibit enzymatic activity. Thus, the role of ultrasound is more dynamic than destructive.

The influence of ultrasonication on the structure of cellulose was basically excluded. Therefore, the key role of transporting substances within cellulose fibres was confirmed. In this region, the action of the longitudinal sound wave, causing local changes in density and pressure, significantly facilitates the transport of molecules. At this stage of research, it is not possible to determine whether facilitating the transport of enzymes is more important due to the increased available surface area of cellulose or removing hydrolysis products from the reaction environment outside the fibre, which can act as enzyme inhibitors. Ultrasound offers both opportunities and limitations in enzymatic hydrolysis. It can significantly improve reaction kinetics when used for a short time. However, prolonged exposure negatively affects enzyme stability, reducing overall efficiency. This study emphasises the crucial role of timing and process control in integrating ultrasound into enzymatic hydrolysis processes. While ultrasound alone cannot overcome the natural resistance of untreated wood, it shows great promise for pretreatment or purification systems. In future applications, combining short ultrasonication with enzymatic hydrolysis and pretreatment methods may provide the optimal balance—increasing speed without sacrificing efficiency.

It is important to summarise that the final glucose yields observed in ultrasound-assisted enzymatic hydrolysis are the result of a complex interplay of multiple, often competing, physical and biochemical phenomena induced by ultrasonication. Cavitation, microstreaming, transient pressure fluctuations, and localised temperature spikes can all occur simultaneously during ultrasound treatment, each affecting different aspects of the hydrolysis process. These effects may enhance enzyme accessibility and mass transfer but can also lead to partial enzyme deactivation or substrate reorganisation, depending on the ultrasound intensity, duration, and the physicochemical nature of the biomass.

## 4. Conclusions

In the case of native wood substrates, as demonstrated in this study, the continuous application of ultrasound during enzymatic hydrolysis did not improve glucose yields for either *P. trichocarpa* or *P. deltoides* × *maximowiczii*. The limited efficacy of ultrasound on untreated woody biomass is due to the protective and rigid structure formed by lignin and hemicellulose, which restricts enzyme access to cellulose.

In contrast, our experiments with cellulose isolated from wood revealed a different behaviour. Here, ultrasonication significantly enhanced the initial rate of enzymatic hydrolysis, particularly in the early stages of the reaction. The effect was especially pronounced within the first few hours, suggesting that ultrasound facilitates mass transfer phenomena, such as enzyme diffusion into the porous cellulose matrix and removal of hydrolysis products, both of which can otherwise inhibit enzyme activity if they accumulate. However, prolonged ultrasonication ultimately resulted in a reduction in glucose yield, most likely due to enzyme deactivation. Direct enzyme activity measurements confirmed it and are consistent with literature reports indicating protein denaturation or oxidative damage from radicals and shock waves generated during ultrasound exposure.

Ultimately, while ultrasound alone does not overcome the recalcitrance of native wood, it remains a promising process intensification tool when integrated with proper pretreatment strategies. Its effects on mass transfer, enzyme diffusion, and potentially even substrate reorganisation at the microscopic level may provide key advantages in well-designed biomass conversion workflows. Future studies should aim to optimise ultrasound exposure time, frequency, and power levels, particularly in combination with pretreatment techniques, to harness its potential in large-scale bioconversion systems fully.

## Figures and Tables

**Table 1:** Glucose yield (±SD) after 72 h enzymatic hydrolysis with and without ultrasonic irradiation of poplar wood of 3-year-old *P. trichocarpa* and 5-year-old *P. deltoides* × *maximowiczii*.

	3-Year-Old *P. trichocarpa*	5-Year-Old *P. deltoides* × *maximowiczii*
without US	(10.1 ± 0.8)%	(9.9 ± 0.3)%
with US	(10.0 ± 0.8)%	(8.1 ± 1.2)% *

* Statistically significant differences (control group), based on the *t*-test (*p* ≤ 0.05).

**Table 2:** The activity of Cellic^®^ CTec2 enzyme blend (±SD) after 4 h of ultrasound treatment at 50 °C, heat treatment in a water bath at 50 °C, and untreated.

Treatment Type	Enzyme Blend Activity/(FPU/cm^3^)
none	129 ± 6.0
50 °C for 4 h	127 ± 5.6
ultrasounds 40 kHz at 50 °C for 4 h	93 ± 1.7 *

* Statistically significant differences (control group), based on the *t*-test (*p* ≤ 0.05).

**Table 3:** The effect of temperature and ultrasound on TCI (±SD) of cellulose.

Pretreatment Type	Time	TCI
reference	0	0.31 ± 0.03
T	0.5	0.35 ± 0.03
	1	0.33 ± 0.03
	2	0.36 ± 0.04
	4	0.34 ± 0.03
	8	0.34 ± 0.04
	24	0.32 ± 0.05
US	0.5	0.34 ± 0.01
	1	0.35 ± 0.03
	2	0.34 ± 0.03
	4	0.37 ± 0.03 *
	8	0.35 ± 0.02 *
	24	0.36 ± 0.03 *

* Statistically significant differences (control group), based on the *t*-test (*p* ≤ 0.05).

**Table 4:** The effect of temperature and ultrasound on LOI (±SD) of cellulose.

Pretreatment Type	Time/h	LOI
reference	0	1.56 ± 0.14
T	0.5	1.62 ± 0.09
	1	1.60 ± 0.10
	2	1.60 ± 0.07
	4	1.60 ± 0.06
	8	1.58 ± 0.04
	24	1.54 ± 0.07
US	0.5	1.60 ± 0.07
	1	1.61 ± 0.06
	2	1.59 ± 0.03
	4	1.51 ± 0.05
	8	1.56 ± 0.10
	24	1.59 ± 0.06

There were no statistically significant differences (control group), based on the *t*-test (*p* ≤ 0.05).

## Data Availability

The original contributions presented in this study are included in the article. Further inquiries can be directed to the corresponding author.

## References

[1] Rosales-Calderon O., Arantes V. (2019). A Review on Commercial-Scale High-Value Products That Can Be Produced alongside Cellulosic Ethanol. Biotechnol. Biofuels.

[2] Yang B., Dai Z., Ding S.-Y., Wyman C.E. (2011). Enzymatic Hydrolysis of Cellulosic Biomass. Biofuels.

[3] Prasad R.K., Chatterjee S., Mazumder P.B., Gupta S.K., Sharma S., Vairale M.G., Datta S., Dwivedi S.K., Gupta D.K. (2019). Bioethanol Production from Waste Lignocelluloses: A Review on Microbial Degradation Potential. Chemosphere.

[4] Álvarez C., Reyes-Sosa F.M., Díez B. (2016). Enzymatic Hydrolysis of Biomass from Wood. Microb. Biotechnol..

[5] Modenbach A.A., Nokes S.E. (2013). Enzymatic Hydrolysis of Biomass at High-Solids Loadings—A Review. Biomass Bioenergy.

[6] Manzanares P. (2020). The Role of Biorefinering Research in the Development of a Modern Bioeconomy. Acta Innov..

[7] Zoghlami A., Paës G. (2019). Lignocellulosic Biomass: Understanding Recalcitrance and Predicting Hydrolysis. Front. Chem..

[8] Pan X., Gilkes N., Saddler J.N. (2006). Effect of Acetyl Groups on Enzymatic Hydrolysis of Cellulosic Substrates. Holzforschung.

[9] Alvira P., Tomás-Pejó E., Ballesteros M., Negro M.J. (2010). Pretreatment Technologies for an Efficient Bioethanol Production Process Based on Enzymatic Hydrolysis: A Review. Bioresour. Technol..

[10] Haghighi Mood S., Hossein Golfeshan A., Tabatabaei M., Salehi Jouzani G., Najafi G.H., Gholami M., Ardjmand M. (2013). Lignocellulosic Biomass to Bioethanol, a Comprehensive Review with a Focus on Pretreatment. Renew. Sustain. Energy Rev..

[11] Ioelovich M., Morag E. (2011). Effect of Cellulose Structure on Enzymatic Hydrolysis. Bioresources.

[12] Torget R., Himmel M.E., Grohmann K. (1991). Dilute Sulfuric Acid Pretreatment of Hardwood Bark. Bioresour. Technol..

[13] Zhu J.Y., Wang G.S., Pan X.J., Gleisner R. (2009). Specific Surface to Evaluate the Efficiencies of Milling and Pretreatment of Wood for Enzymatic Saccharification. Chem. Eng. Sci..

[14] Bay M.S., Karimi K., Esfahany M.N., Kumar R. (2020). Structural Modification of Pine and Poplar Wood by Alkali Pretreatment to Improve Ethanol Production. Ind. Crops Prod..

[15] Kučerová V., Výbohová E., Hönig V., Čabalová I. (2020). Chemical Changes within Solids during Liquid Hot Water Pretreatment of Wood. Bioresources.

[16] Gałązka A., Szadkowski J. (2021). Enzymatic Hydrolysis of Fast-Growing Poplar Wood after Pretreatment by Steam Explosion. Cellul. Chem. Technol..

[17] Maceiras R., Alfonsín V., Seguí L., González J.F. (2021). Microwave Assisted Alkaline Pretreatment of Algae Waste in the Production of Cellulosic Bioethanol. Energies.

[18] Sjulander N., Kikas T. (2022). Two-Step Pretreatment of Lignocellulosic Biomass for High-Sugar Recovery from the Structural Plant Polymers Cellulose and Hemicellulose. Energies.

[19] Szadkowski J., Výbohová E., Kučerová V., Čabalová I., Antczak A., Szadkowska D., Drożdżek M., Zawadzki J. (2024). Effect of Steam Explosion Pretreatment of Fast-Growing Poplar (*Populus deltoides* x *maximowiczii*) Wood on Selected Properties of Structural Substances. Wood Sci. Technol..

[20] Hadar Y. (2013). Sources for Lignocellulosic Raw Materials for the Production of Ethanol. Lignocellulose Conversion: Enzymatic and Microbial Tools for Bioethanol Production.

[21] Xu C., Liao B., Shi W. (2013). Organosolv Pretreatment of Pine Sawdust for Bio-Ethanol Production. Pretreatment Techniques for Biofuels and Biorefineries.

[22] Séguin A. (2011). How Could Forest Trees Play an Important Role as Feedstock for Bioenergy Production?. Curr. Opin. Environ. Sustain..

[23] Putra N.R., Veza I., Irianto I. (2024). Harnessing Wood Waste for Sustainable Biofuel: A Bibliometric Analysis and Review of Valorisation Strategies. Waste Manag. Bull..

[24] Easson M.W., Condon B., Dien B.S., Iten L., Slopek R., Yoshioka-Tarver M., Lambert A., Smith J. (2011). The Application of Ultrasound in the Enzymatic Hydrolysis of Switchgrass. Appl. Biochem. Biotechnol..

[25] Subhedar P.B., Gogate P.R. (2014). Enhancing the Activity of Cellulase Enzyme Using Ultrasonic Irradiations. J. Mol. Catal. B Enzym..

[26] Brennen C.E. (1995). Cavitation and Bubble Dynamics.

[27] Riesz P., Berdahl D., Christman C. (1985). Free Radical Generation by Ultrasound in Aqueous and Nonaqueous Solutions. Environ. Health Perspect..

[28] Menkus J. (2011). Badanie Wpływu Czynników Mechanicznych Na Strukturę Mezoporowatą Drewna Topoli (*Populus* sp.). Ph.D. Thesis.

[29] Bussemaker M.J., Zhang D. (2013). Effect of Ultrasound on Lignocellulosic Biomass as a Pretreatment for Biorefinery and Biofuel Applications. Ind. Eng. Chem. Res..

[30] Tang Siah Ying and Sivakumar M., Fang Z., Smith R.L., Qi X. (2015). Ultrasound as a Green Processing Technology for Pretreatment and Conversion of Biomass into Biofuels. Production of Biofuels and Chemicals with Ultrasound.

[31] Wang Z., Lin X., Li P., Zhang J., Wang S., Ma H. (2012). Effects of Low Intensity Ultrasound on Cellulase Pretreatment. Bioresour. Technol..

[32] Wongjewboot I., Kangsadan T., Kongruang S., Burapatana V., Pripanapong P. (2010). Ethanol Production from Rice Straw Using Ultrasonic Pretreatment. Proceedings of the 2010 International Conference on Chemistry and Chemical Engineering.

[33] Yachmenev V., Condon B., Klasson T., Lambert A. (2009). Acceleration of the Enzymatic Hydrolysis of Corn Stover and Sugar Cane Bagasse Celluloses by Low Intensity Uniform Ultrasound. J. Biobased Mater. Bioenergy.

[34] Revin V., Atykyan N., Zakharkin D. (2016). Enzymatic Hydrolysis and Fermentation of Ultradispersed Wood Particles after Ultrasonic Pretreatment. Electron. J. Biotechnol..

[35] Wang D., Yan L., Ma X., Wang W., Zou M., Zhong J., Ding T., Ye X., Liu D. (2018). Ultrasound Promotes Enzymatic Reactions by Acting on Different Targets: Enzymes, Substrates and Enzymatic Reaction Systems. Int. J. Biol. Macromol..

[36] Kürschner K., Hoffer A. (1929). Ein Neues Verfahren Zur Bestimmung Der Cellulose in Hölzern Und Zellstoffen. Tech. Chem. Pap. Zellst. Fabr..

[37] Nelson M.L., O’Connor R.T. (1964). Relation of Certain Infrared Bands to Cellulose Crystallinity and Crystal Latticed Type. Part I. Spectra of Lattice Types I, II, III and of Amorphous Cellulose. J. Appl. Polym. Sci..

[38] Nelson M.L., O’Connor R.T. (1964). Relation of Certain Infrared Bands to Cellulose Crystallinity and Crystal Lattice Type. Part II. A New Infrared Ratio for Estimation of Crystallinity in Celluloses I and II. J. Appl. Polym. Sci..

[39] Adney B., Baker J. (1996). Measurement of Cellulase Activities; Laboratory Analytical Procedure (LAP).

[40] Wu H., Dai X., Zhou S.-L., Gan Y.-Y., Xiong Z.-Y., Qin Y.-H., Ma J., Yang L., Wu Z.-K., Wang T.-L. (2017). Ultrasound-Assisted Alkaline Pretreatment for Enhancing the Enzymatic Hydrolysis of Rice Straw by Using the Heat Energy Dissipated from Ultrasonication. Bioresour. Technol..

[41] Yang C.-Y., Fang T.J. (2014). Combination of Ultrasonic Irradiation with Ionic Liquid Pretreatment for Enzymatic Hydrolysis of Rice Straw. Bioresour. Technol..

[42] Yu X., Bao X., Zhou C., Zhang L., Yagoub A.E.-G.A., Yang H., Ma H. (2018). Ultrasound-Ionic Liquid Enhanced Enzymatic and Acid Hydrolysis of Biomass Cellulose. Ultrason. Sonochem.

[43] Luzzi S.C., Artifon W., Piovesan B., Tozetto E., Mulinari J., Kuhn G.d.O., Mazutti M.A., Priamo W.L., Mossi A.J., Silva M.F. (2017). Pretreatment of Lignocellulosic Biomass Using Ultrasound Aiming at Obtaining Fermentable Sugar. Biocatal. Biotransform..

[44] Gavrila A.I., Vartolomei A., Calinescu I., Vinatoru M., Parvulescu O.C., Psenovschi G., Chipurici P., Trifan A. (2024). Ultrasound-Assisted Alkaline Pretreatment of Biomass to Enhance the Extraction Yield of Valuable Chemicals. Agronomy.

[45] Szabó O.E., Csiszár E. (2013). The Effect of Low-Frequency Ultrasound on the Activity and Efficiency of a Commercial Cellulase Enzyme. Carbohydr. Polym..

[46] Mawson R., Gamage M., Terefe N.S., Knoerzer K. (2010). Ultrasound in Enzyme Activation and Inactivation. Ultrasound Technologies for Food and Bioprocessing.

